# Elevated Serum Thyroglobulin and Low Iodine Intake Are Associated with Nontoxic Nodular Goiter among Adults Living near the Eastern Mediterranean Coast

**DOI:** 10.1155/2014/913672

**Published:** 2014-12-14

**Authors:** Yaniv S. Ovadia, Dov Gefel, Svetlana Turkot, Dorit Aharoni, Shlomo Fytlovich, Aron M. Troen

**Affiliations:** ^1^Department of Internal Medicine “C”, Barzilai Medical Center Ashkelon, Hahistadrout Street 2, 7830604 Ashkelon, Israel; ^2^Nutrition and Brain Health Laboratory, School of Nutritional Sciences, Institute of Biochemistry, Food Science and Nutrition, Robert H. Smith Faculty of Agriculture, Food and Environment, The Hebrew University of Jerusalem, P.O. Box 12, 76100 Rehovot, Israel; ^3^Endocrinology Clinic, Barzilai Medical Center Ashkelon, Hahistadrout Street 2, 7830604 Ashkelon, Israel; ^4^Laboratory of Clinical Biochemistry, Barzilai Medical Center Ashkelon, Hahistadrout Street 2, 7830604 Ashkelon, Israel

## Abstract

*Background.* Information about iodine intake is crucial for preventing thyroid diseases. Inadequate iodine intake can lead to thyroid diseases, including nontoxic nodular goiter (NNG).* Objective.* To estimate iodine intake and explore its correlation with thyroid diseases among Israeli adults living near the Mediterranean coast, where iodine-depleted desalinated water has become a major source of drinking water.* Methods.* Cross-sectional study of patients attending Barzilai Medical Center Ashkelon. Participants, who were classified as either NNG (*n* = 17), hypothyroidism (*n* = 14), or control (*n* = 31), provided serum thyroglobulin (Tg) and completed a semiquantitative iodine food frequency questionnaire.* Results.* Elevated serum Tg values (Tg > 60 ng/mL) were significantly more prevalent in the NNG group than in the other groups (29% versus 7% and 0% for hypothyroidism and controls, resp., *P* < 0.05). Mean estimated iodine intake was significantly lower in the NNG group (65 ± 30 *μ*g/d) than in controls (115 ± 60 *μ*g/d) (*P* < 0.05) with intermediate intake in the hypothyroid group (73 ± 38 *μ*g/d).* Conclusions.* Elevated serum Tg values and low dietary iodine intake are associated with NNG among adult patients in Ashkelon District, Israel. Larger studies are needed in order to expand on these important initial findings.

## 1. Introduction

Either high or low iodine intake can lead to thyroid disease (TD) [1]. Thus, assessment of iodine intake is crucial for TD prevention. One form of TD is nontoxic nodular goiter (NNG). Nontoxic nodular goiter is a benign thyroid enlargement [2] that may cause neck discomfort, respiratory symptoms, or dysphagia and can lead to thyroid dysfunction [2, 3]. Nontoxic nodular goiter is rarely reversible in adults [4, 5]. Hence, consuming the adequate amount of iodine should reduce NNG risk, perhaps decreasing TD incidence [4, 6]. Worldwide, long-term inadequate iodine intake (over months or years) is associated with high NNG rates [4], but whether low or high intake is the major factor seems to depend on geographic region and timing. For example, NNG was attributed to iodine deficiency (ID) in Greece during the 1960s [6], whereas it was associated with excess iodine intake in Algeria during the previous decade [7]. The possibility that ID contributes to NNG in a developed state as Israel has global significance, given the drop in iodine intake in other industrialized countries in the recent decade [4].

The aim of this study was therefore to investigate the relationship between iodine intake and the presence of TD among Israeli adults, both inpatients and outpatients, within a single region (Ashkelon District). There is limited information about the extent of Israelis harboring TD and there are no national data on iodine intake; however, data from the first and second Israeli National Health Interview Surveys (INHIS-1) [8] (INHIS-2) [9] by Israel Center for Disease Control (ICDC) show that, in 2003–2010, the self-reported use of TD medication among Israeli adults increased from 2.9% to 4.7% (personal communication, ICDC 2013). Our literature review yielded only one study that found no correlation between iodine intake and NNG among Israeli adults (Benbassat et al. 2004) [10]. However, since then iodine-depleted desalinated water has become a major source of drinking water nationwide, particularly throughout the Ashkelon District (personal communication with Mr. Farkash, Israel's South Region Water Quality Engineer, 2011) [11].

Serum thyroglobulin (Tg) has been used as a sensitive marker for ID in many populations studies, and it positively correlates with ID severity as well as with some types of TD, including NNG [12, 13]. Furthermore, because serum Tg falls rapidly with iodine repletion, it is considered a more sensitive indicator of iodine repletion than serum thyrotropin (TSH) or free thyroxine (FT4) [13]. Therefore, we evaluated iodine intake by measuring serum Tg.

In addition to serum Tg values, we estimated habitual dietary iodine intake over the past year using a validated, semiquantitative, Iodine Food Frequency Questionnaire (sIFFQ) that we adapted to Israel. Although this sIFFQ does not include all foods consumed daily, it covers selected iodine-rich foods that provide the majority of dietary iodine, while the remaining food groups probably contain only about 3–6% of the total iodine amount consumed per day [14]. The approach presented here is sound in that it combines Tg and sIFFQ, two complementary indicators: Tg indicates intermediate (weeks to months) iodine intake [6], and the sIFFQ can classify longer-term (up to a year) intake to high and low intake [14]. Although urinary iodine concentration (UIC) is a common measure in many population studies [6, 7, 10], it was not suitable for this study, because of the high within-individual variability in UIC and because UIC indicates recent iodine intake (days), whereas NNG develops after long-term iodine inadequacy in the order of years [15].

## 2. Material and Methods

### 2.1. Settings

**Figure 1:**
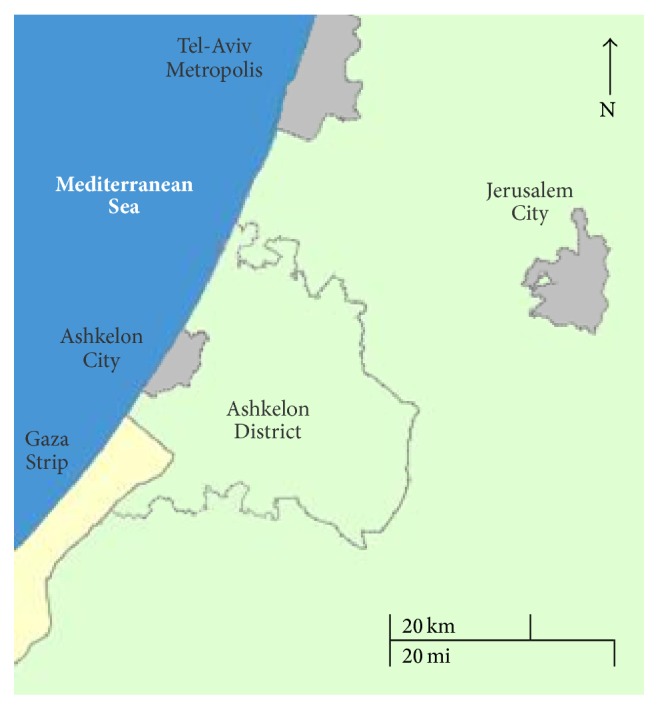
Map of Israel southern coastal area, showing Ashkelon District territory and location (reproduced with permission from The Israel Central Bureau of Statistics: http://gis.cbs.gov.il/benyam/).

In this cross-sectional study, iodine status was assessed in a convenience sample of volunteers living in the Ashkelon District, Israel, who were prospectively recruited at Barzilai Medical Center in Ashkelon (BMCA). The Ashkelon District and city, where the BMCA is located, are shown in the accompanying map (Figure 1). Volunteers were enrolled over a period of 22 months (from March 2012 to January 2014).

### 2.2. Participants

The research was approved by the BMCA Medical Ethics Committee. All participants provided written informed consent after the protocol was carefully explained to them.

Participants included 62 euthyroid Jewish Caucasian adults (50 women, 12 men), aged 21–80. These participants were outpatients attending the endocrine clinic, inpatients, and five hospital workers at BMCA. Every participant's medical file was carefully screened and all volunteers were interviewed with the aid of structured sociodemographic, health, and habits questionnaires. These questionnaires were based on previously validated governmental materials from the Israeli National Nutrition and Health Survey (MABAT) [16]. A series of multiple-choice and open-ended questions were used to collect information on demographic characteristics that could influence dietary and lifestyle practices. Self-reported health status and knowledge, attitudes, and behaviors regarding nutrition and health were also included in these questionnaires. Only volunteers who did not change their address or iodine intake habits (during the two years prior to initiating this study) were included in this study, as were those with BMI of 18.5–35 (kg/h^2^), non-iodine-containing or steroidal drugs consumption, and without any past or current cancer diagnosis nor pregnancy. We also limited the study to those whose first diagnosis of any TD, by both medical file screen and self-reported TD status, took place less than five years prior to the study.

All participants were asked about TD risk factors. These included (a) daily dietary goitrogen exposure and a series of relevant items derived from MABAT [16] and INHIS-2 [9], including (b) previous X-ray examinations involving the jaw or neck area, (c) family history of TD, (d) current smoking habits, and (e) menopause (for women).

The diagnostic criterion for NNG was untreated euthyroid goiter consisting of a single benign nodule or multiple benign nodules. Each nodule was diagnosed as benign according to a fine needle aspiration (FNA) biopsy report. Hypothyroidism was diagnosed when past serum TSH higher than 4 mIU/L was first reported, within five years prior to initiating this study, with overt or subclinical hypothyroidism with or without Hashimoto thyroiditis. Detailed diagnostic criteria for all thyroid conditions included in this study are listed in Table 1.

### 2.3. Assays

Blood samples were taken from all participants without prior fasting and within 24 hours of the participant's signing the informed consent form. Samples were centrifuged immediately and serum was separated and stored at −20°C; serum values of TSH, FT4, thyroid peroxidase antibody (TPOAb), thyroglobulin antibody (TgAb), and Tg were measured for all participants, using the IMMULITE 2000 analyzer (Immunometric Chemiluminescent Assay (ICLA), Siemens Healthcare Diagnostics, Llanberis, UK; the Tg intra-assay CV 4.8%–6.8%, interassay CV 5.6%–10.0%).

Ranges for normal thyroid tests were TSH: 0.4 to 4 mIU/L; FT4: 0.8 to 1.8 ng/dL, according to the National Academy of Clinical Biochemistry [17]. Values of TPOAb above 35 IU/mL were considered positive [18]. Each volunteer with positive TgAb (>40 IU/mL) [18] was excluded. Serum Tg was detectable and available among all participants. Values of serum Tg above 60 ng/mL were considered abnormally elevated by the Laboratory of Clinical Biochemistry at BMCA.

### 2.4. Estimated Iodine Intake

The sIFFQ was designed to estimate participants' daily iodine intake during the one-year period prior to the administration of the questionnaire. This sIFFQ outline was adapted and translated from similar validated questionnaires [14, 19] with local modifications by one of the authors (YSO), a trained Registered Dietitian. A group of experts reviewed the sIFFQ instrument. The group included professional dietitians with experience working with in- and outpatients who were knowledgeable concerning the construction of validated questionnaires. The sIFFQ was pilot tested in a representative sample of both in- and outpatients for readability, clarity of instruction, ease of administration, and time needed for completion. It contained questions regarding the average frequency and amounts of 25 selected foods with relatively high iodine content which Israelis generally consume. The interview was conducted by a single RD (YSO) to minimize variability. Food models, measuring tools, and photographs were provided, when necessary, in order to reduce variation among interviewees. Participants were not informed that the purpose of the interview was to estimate iodine intake. Follow-up phone calls to interviewees were made to clarify information, as, for example, when an interviewee had to check the manufacturer's label for iodine amounts in a dietary supplement. The mean estimated daily dietary intake level was calculated as follows:

(1) $$\begin{array}{c} I=\sum F_{i}\times Q_{i}\times C_{i}, \end{array}$$

where *I* is iodine intake, ∑ is the amount or number of food items, *F* is the frequency per day, *i* represents food items, *Q* is the quantity or serving size, and *C* is the iodine content in the food.

The content of iodine in the participants' food was derived from multiple sources: specific food items from the Department of Nutrition at the Ministry of Health (iodine composition investigation in food by Dr. Eli Havivi 1989); fresh water fish from the Agricultural Service of Israel and the Israeli Fish Breeders Association (fresh water fish nutritional composition, 2012); tap drinking water from the Department of Environmental Health, Public Health Service at the Ministry of Health (survey of iodide levels of drinking water in the Ashkelon District by Israeli Drinking Water National Engineer, Mrs. Irit Hen, 2008); and other sources [10, 20–22].

### 2.5. Statistical Analysis

All statistical analyses were performed with JMP software (Version  7), except for prevalence of elevated serum Tg values for which a Fisher exact test was performed with SAS software (Version  9). A chi-square test was used to compare the prevalence of TD risk factors in the three groups. Age and estimated dietary iodine intake results are expressed as mean ± standard deviation (SD). Comparison of age, mean serum Tg values, and estimated mean dietary iodine intake for the three groups was performed by ANOVA, followed by pairwise comparisons using the Tukey-Kramer HSD method (*α* = 0.05). Data of serum Tg values were log-transformed before analysis in order to stabilize variances. Mean and median of serum Tg were calculated on transformed data.

## 3. Results

The three study groups were similar in age and gender distribution, as well as in socioeconomic status. In addition, the prevalence of known risk factors for TD did not differ significantly between groups.

### 3.1. Serum Tg Values

A comparison of serum Tg concentrations between groups found a significant difference between the NNG and hypothyroidism groups (*P* < 0.05). The prevalence of elevated serum Tg values (Tg > 60 ng/mL) was significantly higher among the NNG group than in the other groups (29% versus 7% and 0% for hypothyroidism and controls, resp., *P* < 0.05) and the mean Tg concentration among NNG group (35 ng/mL) was significantly higher than in the hypothyroidism group (11 ng/mL).

### 3.2. Iodine Intake Estimation by the sIFFQ

At 65 ± 30 *μ*g/d, the mean estimated iodine intake of the NNG group was 42% lower than that of controls (115 ± 60 *μ*g/d) with intermediate intake among hypothyroid patients (73 ± 38). Comparisons for all pair groups showed that these intakes differed significantly between the NNG group and the control group (*P* < 0.05).

**Figure 2:**
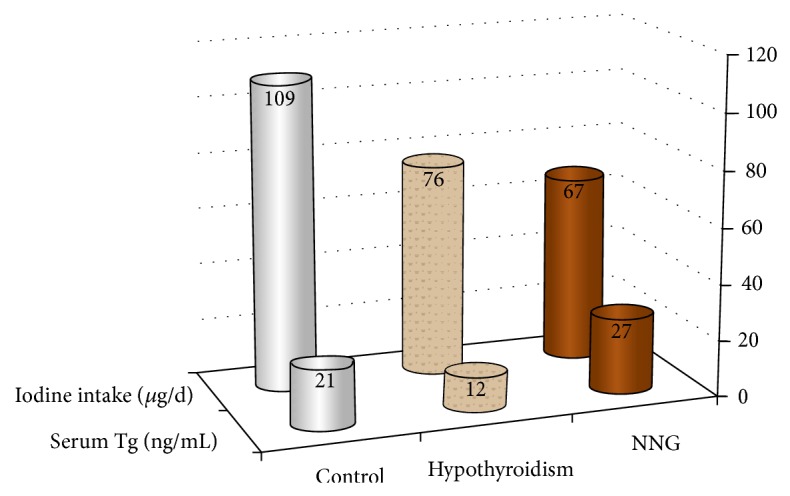
Medians of estimated dietary iodine intake and serum Tg by group. Numbers at the top of each column are median values. Tg: thyroglobulin; NNG: nontoxic nodular goiter; ng/mL: nanogram per milliliter; *μ*g/d: microgram per day.

A comparison by group of detailed mean, median, range, and prevalence of elevated serum Tg, as well as estimated iodine intake by sIFFQ, is given in Table 2. Median estimated dietary iodine intake and serum Tg are shown by group in Figure 2.

## 4. Discussion

In this study, we found a higher prevalence of elevated serum Tg values, as well as lower mean dietary iodine intake among NNG patients as compared with euthyroid controls and hypothyroid patients. Significantly increased serum Tg values among NNG patients compared with the controls have been reported in a comparable epidemiologic study in a Mediterranean population from Sicily, Italy [23]. As in that study, our present findings show the same general trend of elevated Tg among NNG patients compared to controls; however, our sample size is probably too small to reach statistical significance.

Our present results of a nonsignificant difference in Tg values between the hypothyroidism group and the controls resembled a similar Saudi study, among Jeddah area dwellers [19]. This apparent pattern may indicate that elevated serum Tg values are more closely linked with NNG than to hypothyroidism.

Serum Tg values of the NNG group in the current study (mean, median, and 25th–75th percentiles range: 35, 27, and 10–182 ng/mL) were higher than those of both NNG patients' groups in similar small-scale studies: in the greater area of Thessaloniki, Greece (median, 25th–75th percentiles range: 31, 13–70 ng/mL) [24], and in Sicily (mean, range: 34, 0–111 ng/mL) [23]. Destruction of the thyroid follicles and release of Tg in the circulation within 10–15 days after FNA have been suggested to cause serum Tg elevation [24, 25]. Nevertheless, none of the present studied blood samples was taken during 15 days after FNA. Therefore, the contrast between the low iodine intake in the present study and the habitual consumption of iodized salt and bread in Thessaloniki (personal communication, Athanasios D. Anastasilakis, Department of Endocrinology, 424 Military Hospital, Thessaloniki, 2014) and the iodine sufficiency known for Catania city area, Sicily [23], can explain our relatively higher serum Tg values.

The mean dietary iodine intake among NNG participants (65 ± 30 *μ*g/d) was estimated as significantly lower than among controls (115 ± 60 *μ*g/d) and both are much lower than the RDA, which is 150 *μ*g/d [26]. These findings contradict the postulate that because Israel is on the Mediterranean it is an iodine-sufficient country [27]. Moreover, our present findings of low iodine intake among the overall sample and in the NNG group in particular differ from those previously reported about a decade ago by Benbassat et al. [10]. One explanation for this finding can be the increased use of iodine-depleted desalinated water in recent years as the dominant source of drinking water in the Ashkelon District [11]. Another explanation may be related to the fact that, in our study, unlike that of Benbassat et al. [10], we excluded patients who had received a diagnosis of NNG more than five years before study. Such exclusion decreases the likelihood of changes in dietary habits between the diagnosis of incident thyroid disease and the subsequent estimation of habitual dietary iodine intake. The time frame we used is appropriate for the natural history of acquired iodine deficiency disorders such as NNG.

## 5. Conclusions

The data presented in this study suggest that elevated serum Tg values are more prevalent among NNG adult patients in Ashkelon District, Israel. In addition, long-term low dietary iodine intake, rather than high intake, is associated with NNG among this population. It appears that proximity to a salt-water sea in and of itself cannot protect against ID and NNG. As this study comprises a relatively small sample, it cannot be extrapolated to the entire Israeli population. However, if, as it suggests, iodine intake is deficient in the population, then this must be urgently investigated in larger-scale studies. Such reinforcement may help to prevent TD in the future.

## Figures and Tables

**Table 1:** Diagnostic criteria for thyroid diseases.

Thyroid disease	Diagnostic criteria
Nodular goiter	
Single nodule	Goiter with nodule >5 mm in diameter
Multiple nodules	≥2 nodules >5 mm in diameter
Hypothyroidism	
Overt	Serum TSH > 4 mIU/L, FT4 < 0.8 ng/dL
Subclinical	Serum TSH > 4 mIU/L, FT4 within the normal range^*^
With Hashimoto thyroiditis	TPOAb > 35 IU/mL with or without nodular goiter (with overt or subclinical hypothyroidism)

^*^The reference range for FT4 is 0.8 to 1.8 ng/dL; to convert values for FT4 from ng/dL to pmol/L, multiply by 12.87.

ng/dL: nanogram per deciliter; pmol/L: picomoles per liter; mIU/L: milli-international units per liter; FT4: free thyroxine; TPOAb: thyroid peroxidase antibody; TSH: thyrotropin.

**Table 2:** Demographic characteristics, serum Tg values, and estimated dietary iodine intake of the study groups.

Characteristics	Control	Hypothyroidism	NNG
*N*	31	14	17
Gender (women, men)	25, 6	12, 2	13, 4
Age (years)			
Mean^±^	58 ± 13	54 ± 17	60 ± 10
Range (years)	23–77	21–71	49–80
Serum Tg (ng/mL)			
Mean	17^AB^	11^B^	35^A^
Median	21	12	27
Range	2–59	1–101	2–792
25th–75th percentiles range	12–32	3–43	10–182
*n* (abnormally elevated values)^P∗^	0 (0%)	1 (7%)	5 (29%)
Estimated dietary iodine intake (*μ*g/d)			
Mean^±^	115 ± 60^B^	73 ± 38^AB^	65 ± 30^A^
Median	109	76	67
Range (*μ*g/d)	27–263	29–165	10–113

^±^Plus-minus values are mean ± SD.

^AB^Means without a common letter are significantly different (Tukey-Kramer, *α* = 0.05).

*n* (abnormally elevated values) = number of participants with Tg values above 60 ng/mL.

^P^Prevalence displayed as number of positive cases (percentage in brackets).

^*^Groups are significantly different (Fisher exact test, *P* < 0.05).

Tg: thyroglobulin; NNG: nontoxic nodular goiter; ng/mL: nanogram per milliliter; *μ*g/d: microgram per day.
